# Acute Liver Failure Secondary to Metastatic Medullary Thyroid Cancer

**DOI:** 10.1155/2011/603757

**Published:** 2012-01-15

**Authors:** Emmanuel C. Gorospe, Jemilat Badamas

**Affiliations:** Saint Marys Hospital, Mayo Clinic, 1216 Second Street SW, Rochester, MN 55902, USA

## Abstract

Acute liver failure (ALF) is a rare presentation of liver metastases. Although cases of ALF from metastatic disease have been reported, etiologies have been largely confined to lymphoma, metastatic breast, lung, and gastric cancers. ALF from medullary thyroid cancer (MTC) has never been reported. We present a 59-year-old male with newly diagnosed MTC, who was admitted with ALF. He presented with jaundice, hepatic encephalopathy, and synthetic dysfunction. His clinical course was marked by rapid decompensation within 6 days from initial presentation of jaundice to development of hepatic coma. Although liver metastases from medullary thyroid cancer have been reported, to our knowledge, this is the first described case of MTC resulting in acute liver failure.

## 1. Introduction

Acute liver failure (ALF) is a critical condition with significant morbidity and mortality. It is characterized by abrupt onset of jaundice, coagulopathy, and hepatic encephalopathy, in the absence of preexisting liver disease [1]. Liver failure is classified as acute if encephalopathy occurs less than 8 weeks after the onset of jaundice [2]. ALF may be further categorized as hyperacute if the interval between onset of jaundice to encephalopathy is less than 7 days [3]. The natural course of ALF proceeds with rapid liver dysfunction, leading to multiorgan failure and death. Its overall mortality rate is 85% [2]. Fortunately, ALF is an uncommon condition, with 1,600 cases of ALF in the USA occurring annually [4]. Leading causes of ALF include acetaminophen-induced liver failure and viral hepatitis [5]. Other reported causes include idiosyncratic drug reactions, toxin-related hepatitis, autoimmune liver disease, acute liver ischemia, and other miscellaneous conditions [2]. Due to its rarity of occurrence, heterogeneity in etiology, and rapidity of onset, ALF remains a difficult syndrome to study.

There is increasing incidence of thyroid cancer in the United State estimated at 7.7 per 100,000 person-years [6]. These cases are mostly of the well-differentiated type with MTC accounting for only 5% of all thyroid neoplasms [6, 7]. MTC is a rare calcitonin-secreting tumor derived from the parafollicular C cells of the thyroid [8]. The survival of MTC patients is between that of well-differentiated and anaplastic thyroid cancers with poor outcome.

ALF from malignant infiltration is uncommon. It is even rarer when it occurs secondary to metastatic medullary thyroid cancer (MTC). Although the liver is a common site of cancer metastasis, we found no published reports of ALF from metastatic MTC after conducting a systematic search of MEDLINE and EMBASE using the terms *acute liver failure*, *fulminant hepatic failure*, *metastasis*, and *medullary thyroid cancer* from database inception to December 2011. Similarly, no report of ALF was found after hand searching the few published case reports of liver metastasis in MTC.

## 2. Case Report

**Figure 1:**
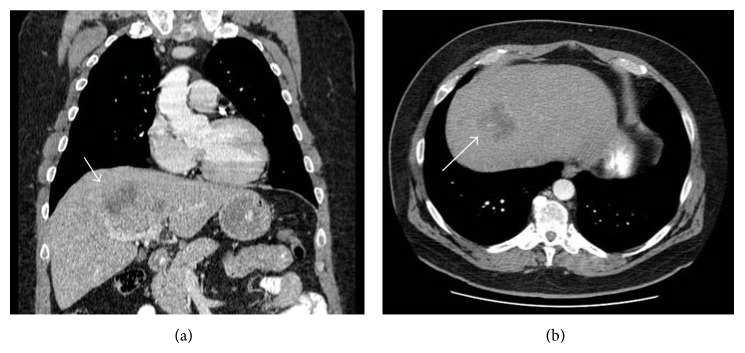
Abdominal CT scan showing hypodense lesion near the dome of the right hepatic lobe (arrow).

**Figure 2:**
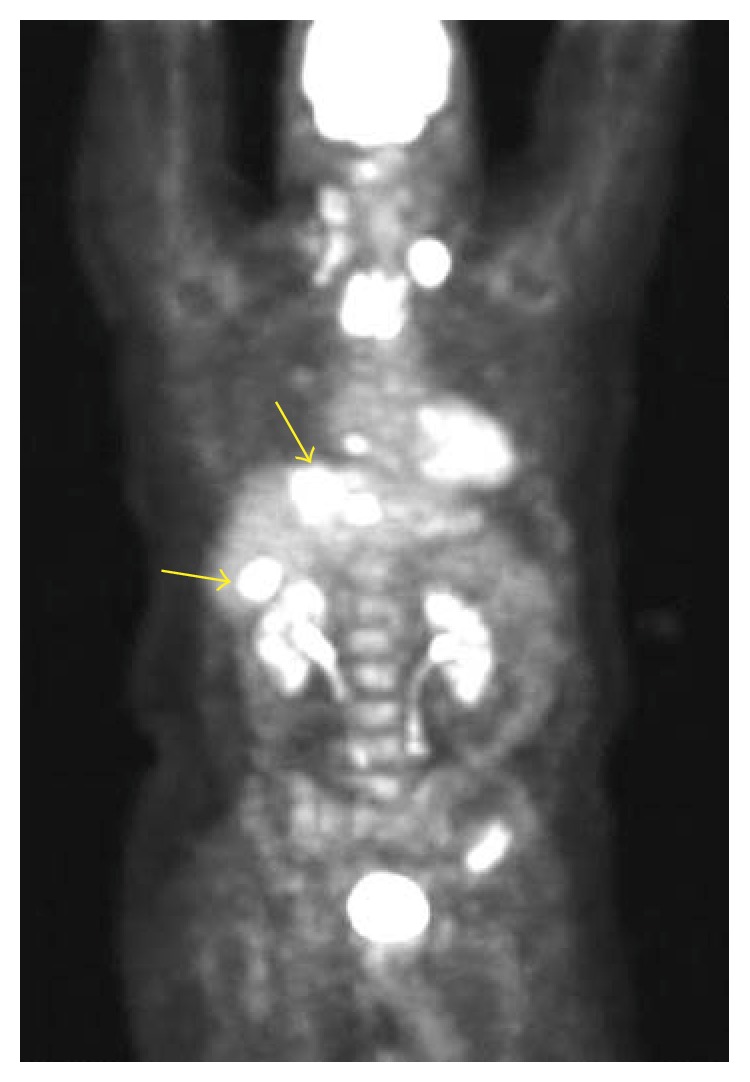
PET scan showing extensive uptake in the neck, chest and liver (arrows).

A previously healthy 59-year-old male without any prior medical history or medication use, initially complained of a five-month history of bilateral neck pain. Outpatient workup at an outside hospital revealed palpable cervical lymph nodes. The excisional biopsies with immunohistochemical staining were consistent with MTC. The patient had no history of alcohol, tobacco, or illicit drug use. He was a school teacher without any history of recent travel or occupational chemical exposure. He had no prior head and neck radiation or family history of thyroid cancer. Screening for the RET (multiple endocrine neoplasia-2) gene mutation was negative. CT scan of the neck showed heterogeneous mass in the right lobe of the thyroid and isthmus with bilateral nodal metastases. His calcitonin level was markedly elevated at 4,402 pg/mL (normal range: 0–19 pg/mL). Carcinoembryonic antigen (CEA) was increased to 9,483 ng/mL (normal range: 0–5 ng/mL). Thyroid-stimulating hormone (TSH) was 2.5 *μ*U/mL (normal range: 0.4–5 *μ*U/mL). Abdominal CT revealed multiple hypodense lesions in the liver. The largest lesion was in segment 8, which measured 5.3 × 4.4 cm (Figure 1). Staging PET scan showed foci of intense uptake of radiopharmaceutical F-18 FDG in the neck, chest, and liver. The largest focus was near the dome of the right hepatic lobe, corresponding with the same area identified in the CT scan (Figure 2). His liver biochemical tests were normal with alanine aminotransferase (ALT) 36 U/L (normal range: 10–45 U/L), aspartate aminotransferase (AST) 58 U/L (normal range: 12–31 U/L), alkaline phosphatase 114 U/L (normal range: 98–251 U/L), total bilirubin 0.4 mg/dL (normal range: 0.1–1.1 mg/dL), and albumin 4.4 g/dL (normal range: 3.5–5 g/dL). The PT INR was 1.1.

The patient underwent primary tumor debulking with a right thyroidectomy and bilateral neck dissection at an outside hospital. Histopathologic examination from the thyroidectomy specimen confirmed MTC. The carcinoma extended extrathyroidally to involve adjacent skeletal muscle and adipose tissue, with all dissected cervical lymph nodes positive for metastases. Three weeks after thyroidectomy and before planned chemotherapy or radiation, the patient acutely developed jaundice, confusion, and fever. Three days after the onset of symptoms, he presented to an outside hospital. Because of concern for acute liver failure, he was immediately transferred to our institution. On admission, the patient's vital signs were stable. He was awake but disoriented with asterixis. He had no stigmata of chronic liver disease except for hepatomegaly with the liver edge palpable 2 cm beyond the right costal margin. There was no flank or shifting dullness, fluid wave, or bipedal edema. Laboratory evaluation revealed a hematocrit of 38.8%, platelets 91,000/cm^3^, leukocytes 22,500/cm^3^, albumin 2.8 g/dL, total bilirubin 10.6 mg/dL, direct bilirubin 6 mg/dL, AST 310 u/L, ALT 186 u/L, alkaline phosphatase 335 u/L, INR 2.8, plasma ammonia 128 *μ*moL/L, and lactic dehydrogenase 920 u/L. The blood cultures from the outside hospital were negative. Tests for hepatitis A, B, and C, toxicology panel, and autoimmune markers were negative. His only home medications were a daily multivitamin and fentanyl 25 mcg patch. Abdominal ultrasound with Doppler evaluation showed increased heterogeneity of the liver with focal lesions, consistent with progression of hepatic metastases. There was no evidence of portal or hepatic vascular occlusion, biliary obstruction, gall bladder disease, or other intra-abdominal pathology. In spite of aggressive antibiotic coverage, lactulose, and rifaximin administration, the patient's mental status persistently deteriorated within a few days of hospitalization. In view of the worsening prognosis, the patient's family decided to proceed with palliation and end-of-life care. The patient developed hepatic coma, shock, and multiorgan failure. He expired on day 4 of admission. Postmortem examination was declined by the family.

## 3. Discussion

Our patient presented a rare course of metastatic MTC, resulting in acute liver failure. The patient's jaundice, rapid encephalopathy, and shock represent a natural progression of ALF with poor outcome. In a series of patients with ALF secondary to malignant hepatic infiltration at King's College Hospital, overall mortality rate was 94% [5]. In the same series, the leading causes of malignant hepatic infiltration were non-Hodgkin's lymphoma and Hodgkin's disease. Other commonly reported malignancies predisposing to liver metastases include breast cancer, gastric carcinoma, small-cell lung cancer, pancreatic cancer, melanoma, and leukemia [5, 9–11]. In metastatic liver disease, the elevation of aminotransferase levels and alkaline phosphatase is the most useful marker [12]. In our patient, the elevated calcitonin levels and absence of other etiologies of ALF suggest metastatic liver disease from MTC. The previously normal liver function tests do not rule out significant hepatic involvement as the degree of liver test dysfunction may not always correlate with the extent of hepatic tumor burden. Modestly elevated liver function tests have been observed in patients with ALF in spite of complete hepatic parenchymal replacement by infiltrating malignancy [11].

The underlying pathophysiology of ALF in our patient was likely from rapid replacement of critical liver mass by the metastatic thyroid neoplasm. The mechanism of ALF in metastasis is multifactorial. Experience from other cases reports the following possibilities: (1) parenchymal infarction due to vascular occlusion by tumor thrombi, (2) rapid loss of critical liver cells from replacement by metastatic tumor, and (3) nonocclusive liver ischemia triggered by shock from other contributing causes such as sepsis and circulatory dysfunction [9, 11, 13].

MTC arises in both familial and sporadic forms. The familial form is associated with multiple endocrine neoplasia (MEN) [7]. In this case, the patient did not have any family history of thyroid cancer. His screening for RET mutation was negative. It is very likely that the patient had a sporadic form of MTC. Germline mutations in the RET protooncogene occur in all patients with inherited MEN 2. However, sporadic type of MTC may have occasional somatic RET mutations only [7]. In the United States, the mean age of MTC patients is around 50 years of age [14]. Hereditary or familiar MTC is diagnosed earlier. As seen in the patient, cervical lymphadenopathy is a common manifestation that triggers workup of malignancy.

Liver metastases in MTC may have an initial miliary pattern that makes radiographic detection difficult. Occasionally, even macroscopic liver metastases may be missed [7]. The CT scan of the liver may not reveal the usual, nodular infiltration if there is diffuse intrasinusoidal spread. When correlated with histopathology, this often reveals diffuse malignant infiltration instead of malignant nodular aggregates [5]. Based on a 35-year retrospective case series, the 10-year survival rate of MTC is 85% [15]. MTC metastases typically occur in the bone, mediastinum, lung, lymph nodes, and liver. The reported cases of liver metastases follow an indolent course without any occurrence of acute liver failure [16–19]. The persistence of disease after primary surgical treatment correlates with elevated calcitonin levels [20]. The early occurrence of distant metastasis and elevated calcitonin levels are poor prognostic factors [15].

In summary, we report an unusual case of acute liver failure. Our case strongly implies that metastatic MTC can cause ALF. While a liver biopsy or postmortem examination was not performed, the diagnosis of acute liver failure from malignant infiltration was supported by the extensive hepatic lesions, persistently elevated calcitonin levels, and absence of other etiologies. Metastatic medullary thyroid cancer should be added to the list of possible malignant causes of ALF.
